# Bias and Misrepresentation of Science Undermines Productive Discourse on Animal Welfare Policy: A Case Study

**DOI:** 10.3390/ani10071118

**Published:** 2020-06-29

**Authors:** Kelly Jaakkola, Jason N. Bruck, Richard C. Connor, Stephen H. Montgomery, Stephanie L. King

**Affiliations:** 1 Dolphin Research Center, Grassy Key, FL 33050, USA; 2 Department of Biology, Stephen F. Austin State University, Nacogdoches, TX 75962-3003, USA; jbruck@sfasu.edu; 3 Biology Department, University of Massachusetts Dartmouth, North Dartmouth, MA 02747, USA; rconnor@umassd.edu; 4 School of Biological Sciences, University of Bristol, Bristol BS8 1TQ, UK; s.montgomery@bristol.ac.uk (S.H.M.); stephanie.king@bristol.ac.uk (S.L.K.)

**Keywords:** animal welfare, misrepresentation, orca, killer whale, captivity, brain size, legislation, policy, management, bias

## Abstract

**Simple Summary:**

Creating good animal welfare-related laws, regulations, and policies depends on accurate knowledge. To that end, scientific reviews that explain and contextualize the relevant research can be powerful tools for informing decision-makers, assuming these reviews represent the state of the scientific knowledge accurately and objectively. In this commentary, we examine the major flaws, biases, and misrepresentations of the scientific literature in one such recent review regarding the welfare and care of captive killer whales. Such pervasive problems, in this or any review, make it impossible to determine the true state of knowledge of the relevant issues, and can ultimately result in misinformed, arbitrary, or even harmful decisions about animals and their care.

**Abstract:**

Reliable scientific knowledge is crucial for informing legislative, regulatory, and policy decisions in a variety of areas. To that end, scientific reviews of topical issues can be invaluable tools for informing productive discourse and decision-making, assuming these reviews represent the target body of scientific knowledge as completely, accurately, and objectively as possible. Unfortunately, not all reviews live up to this standard. As a case in point, Marino et al.’s review regarding the welfare of killer whales in captivity contains methodological flaws and misrepresentations of the scientific literature, including problematic referencing, overinterpretation of the data, misleading word choice, and biased argumentation. These errors and misrepresentations undermine the authors’ conclusions and make it impossible to determine the true state of knowledge of the relevant issues. To achieve the goal of properly informing public discourse and policy on this and other issues, it is imperative that scientists and science communicators strive for higher standards of analysis, argumentation, and objectivity, in order to clearly communicate what is known, what is not known, what conclusions are supported by the data, and where we are lacking the data necessary to draw reliable conclusions.

## 1. Introduction

The past few decades have seen a pronounced increase in interest and attention to animal welfare and its management, as evidenced by growing public concern for animals’ quality of life; significantly more research with an explicit animal welfare focus (including a new discipline area of animal welfare science); and ongoing improvements in professional animal care standards and practices (e.g., [1,2,3,4,5,6,7]). As interest in animal welfare has grown, so has the societal conviction that we should strive for the highest standards of animal care, and continually develop ways of improving the welfare of animals in all parts of our lives [3,5,6,8]. High-quality zoos and aquariums typically seek accreditation by professional organizations, such as the Alliance of Marine Mammal Parks and Aquariums (AMMPA), the Association of Zoos and Aquariums (AZA), the European Association of Zoos and Aquaria (EAZA), and the European Association for Aquatic Mammals (EAAM). The animal welfare-related standards and guidelines of these organizations typically exceed governmental regulations [9] (see, e.g., [10,11,12,13]), but even with such shared high standards, there can be large variation in welfare outcomes [7,14,15]. Moreover, not every facility chooses to participate in these organizations and, therefore, may not meet these same standards. Thus, legislation and regulations are important to ensure welfare protection for all animals in human care [9].

The creation of such laws and regulations is a complex process during which policy-makers must navigate and balance a variety of competing interests, including personal values, political ideology, economic considerations, and practical feasibility [16,17,18]. In addition, informed decision-making, by definition, requires an understanding of the facts relevant to the issue under consideration. In times of emergency, such as a disease outbreak or disaster at a nuclear power plant, governments may convene scientific advisory groups to quickly inform the government’s response [16,19,20]. For other important but perhaps less urgent issues, there are numerous initiatives internationally to create accurate and unbiased syntheses of scientific information to inform policy-makers [16,21]. Such credible scientific assessments of a topic, such as the Global Biodiversity Assessment [22] and the Intergovernmental Panel on Climate Change [23], can be powerful tools to inform policy and management discussions and initiatives [16,21,24].

In recent years, the practice of housing and displaying killer whales (*Orcinus orca*) in marine mammal facilities has received a lot of media attention and lobbying from anti-zoo organizations (e.g., [25,26,27,28]), resulting in proposed legislative bans (e.g., [29,30]). A recent paper in the Journal of Veterinary Behavior [31] purported to review the state of knowledge regarding the welfare of killer whales in captivity, potentially providing a scientific assessment of the kind that could help legislators make informed decisions about this issue. Specifically, Marino et al. [31] argued that, due to the complex needs conferred by their cognitive, emotional, and social characteristics, killer whales cannot thrive in traditional marine mammal facilities. Rather, they argued, captive killer whales exhibit abnormal behaviors and often die at an early age, likely due to chronic stress brought about by captive conditions.

In theory, comprehensive reviews of the research on the physiological, cognitive, behavioral, and social characteristics of a species, along with welfare indicators such as life expectancy, health, stress, behavioral issues, and so forth, should be extremely useful tools for informing discourse on animal welfare-related policy and management activities. Given that they are positioned to impact the legislative and regulatory decisions affecting these animals, it is critical that these assessments strive for an accurate representation of the state of the scientific knowledge about their topic. To do so, they must use sound and rigorous principles of scientific analysis and argumentation, and clearly communicate what is known (and not known) in order to give the public, policy-makers, and legislators the resources they need to participate in constructive dialogue and decision-making [16,21]. Unfortunately, the Marino et al. [31] paper falls short of these standards in a variety of ways, including problematic referencing, overinterpretation of the data, misleading word choice, and fallacious argumentation.

In the current paper, we highlight several of the major methodological flaws, biases, and misrepresentations of the scientific literature in Marino et al.’s [31] review. Note that the specific examples discussed for each issue are not meant to be an exhaustive list of all instances in the paper, but rather are meant to be illustrative of repeated instances throughout their review. Note also that this critique is not intended as an explicit defense of, or argument for, keeping killer whales in captivity. In fact, we (the authors of this paper) hold disagreeing opinions on this topic and will not be taking a stance on this issue one way or the other. However, regardless of our disparate feelings about the underlying issue, we are strongly united in our belief that valid scientific evidence and argumentation is mandatory in order to have an accurate and productive discussion. We therefore offer this critique with the ultimate goal of increasing the level of evidence-based argumentation in the public, scientific, and legislative discourse surrounding this and similar issues.

## 2. Methodological Issues

### 2.1. Problematic Referencing

A review paper is meant to provide a comprehensive summary and synthesis of the scientific literature on a particular topic [32,33]. To do that effectively, there are certain fundamental relations required between the claims made in the paper and the relevant scientific literature. Specifically: (1) Any claims made must be supported by citing scientific references. (2) The references cited must be represented accurately; that is, each reference must say what the authors say it does. (3) The references cited must be representative of the relevant scientific literature; that is, the authors must not “cherry-pick” just those studies or pieces of studies that support their claims, while omitting studies or pieces of studies that disagree with their claims [34]. The paper by Marino et al. [31] repeatedly violates each of these tenets, as discussed below.

#### 2.1.1. Unsupported Claims

There are multiple places where unsupported claims are stated as if they were fact, with no references given. For example:

(a) “*orca calves rely upon their mothers and closely related female relatives within their natal pod for protection, nourishment, and the development of essential life skills that contribute to their long-term psychological health* ….” (p. 75). While the first part of this sentence is quite reasonable, as far as we are aware there have been no studies conducted (and certainly none cited in the Marino et al. [31] review) that measure effects of maternal care on killer whale psychological health, or even provide defensible descriptions of killer whale psychological health.

(b) “… *complex vocal acquisition, and dialect development are examples of social-cognitive-communicative needs found in orcas that are unavailable in concrete tanks*.” (p. 77). This statement is both unreferenced and untrue, as a number of studies have demonstrated that killer whales in marine mammal facilities do learn complex vocalizations from social group members [35,36,37].

#### 2.1.2. Misleading Referencing

There are also a number of places in the Marino et al. [31] paper where the references cited do not in fact support the claims being made. For example:

(a) “*The breadth of available scientific data demonstrates that, by every appropriate metric, captive orcas do not fare as well as their free-ranging counterparts (e.g., Small and DeMaster, 1995; Woodley et al., 1997; Jett and Ventre, 2015; Robeck et al., 2015)*” (p. 69). Despite the claim of “every appropriate metric”, all of the references cited discuss only a single metric of life expectancy [38,39,40,41]. There are no references regarding any additional welfare metrics, such as body condition, disease, cortisol levels, or behavioral measures (e.g., [42,43,44,45]). We discuss additional problems with Marino et al.’s review of the data on life expectancy later in the paper.

(b) “*Vocal learning requires a substantial degree of social awareness and forms an important component of orcas’ multiple cultural traditions, as well as their identity and sense of security and cooperation in a social group, as it does in many other highly social mammals (Cohen, 2012)*.” (p. 72). The Cohen paper [46] does not discuss social awareness at all, just that vocal learning can be used to develop group identifiers, where the convergence of dialects facilitates cooperation between individuals. Indeed, contrary to Marino et al.’s claim, scientific evidence shows that vocal learning does not necessarily require a “substantial degree of social awareness”. Some songbirds, for example, can learn apparently normal song modeled from a tape recording, with no social partner even present (e.g., [47]). Similarly, the Cohen paper does not mention anything about a “sense of security”. The claim here seems to be that vocally learned dialects make individual killer whales feel secure in their group. While learned dialects are used to broadcast group identity [48], there is no empirical evidence to support the suggestion that it makes them feel secure.

(c) “*The confinement of naturally wide-ranging mammals causes chronic stress (Dawkins, 1998)*” (p. 76). The Dawkins [43] paper never discusses chronic stress, nor claims that confining wide-ranging mammals causes it. Dawkins comes closest to this idea in her discussion of reinforcers, noting that preventing an animal from performing a naturally reinforcing behavior (such as when a small mammal is prevented from seeking cover) may be aversive to that animal. It is possible that Marino et al. may have extended this idea to wide-ranging mammals (and substituted chronic stress for aversion) under the assumption that such ranging must be inherently reinforcing for the animals. However, Dawkins cautions against this exact type of thinking, noting that in the absence of specific experimental data, one cannot use the fact that an animal performs a behavior in the wild (e.g., seeking cover or ranging far) to infer that they need to or want to perform that behavior in captivity, where the functional needs that those behaviors provide (e.g., avoiding predators or finding food) may be provided for in other ways. (See also Section 2.4.4 regarding Marino et al.’s discussion of space.)

#### 2.1.3. Selective Referencing

Finally, there were multiple instances in which Marino et al. either neglected to mention relevant scientific literature that conflicted with their claims, or presented selected evidence from a reference that supported their preferred narrative while omitting opposing or less supportive evidence from that same reference. For example:

(a) “*The mean life expectancy for free-ranging orcas is 46 years for females and 31 years for males (Olesiuk et al., 1990, 2005).*” (p. 73). The Olesiuk et al. papers cited here [49,50] actually report several separate estimates of mean life expectancies for wild killer whales from different populations and time periods. These mean life expectancies range from 30–50.2 years for females and 19–31 years for males. As such, the authors should have presented the full range of life expectancies, not just a subset of the data.

(b) *“In mammals, expansion of the neocortex, which is critical for higher-order functions, occurs through folding of the surface… The “gyrification index,” which compares neocortical surface area to total brain weight, ranges from 2.4 to 2.7 for odontocetes, exceeding the value of 1.75 for modern humans (Ridgway and Brownson, 1984).”* (p. 70). Here, Marino et al. seem to be proffering gyrification (i.e., the amount of folding in the cerebral cortex) as a proxy measure for “higher-order (cognitive) functions”, and note that the gyrification in killer whales and other odontocetes is particularly high among mammals. There are several problematic issues with this account. First, the definition given for gyrification index (GI) is incorrect. GI does not compare cortical area to weight, but rather is a ratio of two areas—specifically, of total cortical surface area (i.e., including the areas inside the folds) compared to the exposed or visible surface area (i.e., not including the area within the folds)—thereby giving a specific measure of the amount of folding (e.g., [51,52,53,54]). Secondly, the numbers provided for the GI of odontocetes and humans are also incorrect, taken not from the Ridgway and Brownson [55] paper as cited, but rather from one of Marino’s earlier papers ([56], uncited in the Marino et al. review). In this earlier work, she calculated these numbers using the erroneous formula of neocortical surface area divided by weight noted above. Finally, Marino et al. fail to note that, after accounting for brain size, GI scales negatively with cortical thickness, because thinner cortices allow for easier folding [53,54]. Thus, for a given brain size, ungulates (e.g., sheep, cows) are more gyrencephalic than primates (e.g., monkeys, apes) due to their lower cortical thickness [54]. Similarly, due to having a particularly thin cortex for their brain size, cetaceans have correspondingly high GIs [52,53,54].

### 2.2. Overinterpretation

In any scientific review, accurate reporting and interpretation of research results is essential in order to effectively inform scientific understanding, which is especially important when such understanding may have implications for public discourse and legislative debate. One impediment to such accuracy is overinterpretation, in which scientists—whether intentionally or unintentionally—bias their interpretations in such a way that results are presented as more favorable than is justified [57,58]. In arguing that killer whales have “complex needs” that cannot be met in traditional zoological facilities, Marino et al. [31] repeatedly overinterpret data on killer whale cognitive and neural characteristics in two ways, namely (1) consistently interpreting behaviors and neural features as indicative of the highest possible cognitive explanation, going well beyond the demonstrated data; and (2) failing to contextualize such behaviors and neural features by noting which other animals also exhibit them.

To be clear, our argument here is not meant to imply that if killer whales were shown to possess the cognitive capacities claimed by Marino et al. [31], then this would mean that zoos or aquaria could not meet their needs. To be sure, providing animals with appropriate cognitive challenge is important for positive welfare [59,60,61]. Such cognitive challenges have been provided in zoological settings in a variety of contexts and forms, including cognitive research, puzzle devices, computer tasks, and “thinking games” (i.e., training of conceptual rules, such as innovate and imitate) [59,61,62,63]. While further research is warranted regarding what counts as an appropriate cognitive challenge for killer whales and other animals, this research and the management practices that follow from it can only be served by a valid and accurate assessment of an animal’s cognitive capacities [60,64,65].

Examples of Marino et al.’s [31] overinterpretation of killer whale cognitive and neural characteristics include the following:

#### 2.2.1. Self-Awareness

Marino et al. [31] argue that killer whales have demonstrated abilities indicative of self-awareness, including mirror self-recognition. As evidence, they note that “*In a study of their responses to mirrors, orcas show contingency checking behavior—a correlate of self-directed responses exhibited by most individuals who demonstrate mirror self-recognition (Delfour and Marten, 2001).*” (p. 71). This explanation is misleading, however, given that contingency checking correlates with self-directed responses in the sense that it is a prerequisite. That is, an animal’s (or human child’s) demonstration of self-recognition typically progresses through three phases [66,67,68]. Upon initial exposure to a mirror, animals typically display social behavior as if they interpret the image in the mirror as another animal. The next phase is contingency checking, in which the animals may engage in highly repetitive or unusual movements, as if checking whether the image in the mirror moves when and how they move. Finally, they begin to engage in self-directed behaviors, such as positioning themselves so they can examine and/or groom parts of themselves that they cannot normally examine without a mirror. This third stage is generally interpreted as evidence of mirror self-recognition. Contingency checking by itself does not demonstrate self-awareness [66,68,69]. In addition, Marino et al. fail to contextualize this issue by omitting mention that several animal species have been claimed to pass the mirror self-recognition task at a more stringent level than what killer whales [70] have demonstrated, including elephants [71], bottlenose dolphins [72], several species of ape (e.g., [73,74,75]), one species of bird [76], and one species of fish [77].

#### 2.2.2. Von Economo Neurons

In arguing for “*dimensions of the orca brain that serve as strong predictors of complex cognitive abilities*” (p. 70), Marino et al. [31] state that recent studies show that the brains of larger cetaceans contain a type of neuron (Von Economo neurons (VENs)) “*which may be involved in social cognition (Allman et al., 2005) and adaptive intelligent behavior (Allman et al., 2005) in mammals*” (p. 70). It is important to note that the reference they provide for this [78] is a paper specifically about human brains, written at a time when VENs had only been found in restricted cortical areas of humans and apes. This specific distribution, together with their higher density in the human cortex, initially suggested that these neurons may play a role in social awareness and intuition [79], as Marino et al. suggest. However, Marino et al. fail to mention over a decade of relevant subsequent research. Specifically, when VENs were next found in the brains of cetaceans [80,81] and elephants [82], this was initially taken as further evidence that they were characteristic of socially complex, large-brained species. However, since that time, VENs have also been found in a wider variety of animal species, including macaque monkeys, pygmy hippopotamus, cow, pig, deer, sheep, horse, manatee, walrus, and common zebra [83,84,85,86,87,88]. Since the presence of VENs is not—as was once believed—restricted to large-brained and/or socially complex species, the view of them has consequently changed. Now their wide phylogenetic distribution suggests that they have emerged repeatedly in a variety of animal groups in response to selective forces that remain unclear [88], but may be related to mechanical forces experienced during cortical gyrification [79,88,89].

### 2.3. Misleading Word Choice

In scientific writing, as in all writing, specific word choice affects the accuracy and clarity of the message [90]. Words have meanings, and specific words create specific impressions in the mind of the reader. In several places throughout their paper, Marino et al. create a misleading impression by using specific word choices that imply the existence of conclusions that have not in fact been demonstrated or supported with evidence. For example, they state that killer whales “*possess one of the largest and most complex brains… However, they are the third most common species of cetaceans kept*” in marine mammal facilities (p. 69, emphasis added). This use of “however” suggests that “complex brains” and quality zoological care are inherently incompatible, when no such conclusion has been demonstrated. Similarly, they note that previous papers have not offered an “*explanation for why captive orcas suffer chronic stress*” (p. 69, emphasis added), even though they never present evidence that captive killer whales in fact do suffer chronic stress (see discussion of stress below).

In addition, Marino et al. repeatedly utilize linguistic qualifiers or hedges to suggest possible facts or conclusions for which no evidence has been presented. For example, they note that “*These impacts may be worsened in captivity*” (p. 76) without presenting evidence that they are; that killer whales’ daily routine “*may lead to anxiety and boredom*” (p. 77) when no such effects have been demonstrated; that they “*may have shared food*” (p. 71) when there is no evidence that they did; and “*One could speculate that…*” (p. 74) when of course one can speculate anything (emphases added).

### 2.4. Fallacious Argumentation

Beyond the difficulties with referencing, interpretation, and word choice pervasive throughout Marino et al.’s [31] review, there were also a number of problems or fallacies that were specific to individual arguments and topics, as described below.

#### 2.4.1. Neuroanatomy

In addition to the claims discussed earlier regarding cortical gyrification and Von Economo neurons, Marino et al. make a number of further claims about how the neurobiological characteristics of killer whales provide evidence of their cognitive capacity and “*act as a guidepost to the kind of environment orcas need to thrive*.” (p. 70). Specifically, they argue that (a) relative brain size (specifically the encephalization quotient (EQ)) predicts cognitive capacity across species, and killer whales belong to a taxonomic group that has the highest EQs among nonhuman animals; (b) the expansion of certain brain areas in cetaceans “*is arguably associated with high-level cognitive and social functions such as attention, prediction, social awareness, and empathy*” (p. 70); and (c) cetacean cytoarchitectonic patterns (i.e., the arrangement of cells in their cortex) “*are also evidence of cetacean behavioral and social complexity*” (p. 70).

These arguments are problematic in at least two respects. First, EQ is typically measured as a deviation between the observed brain size and the “expected” estimate of brain size derived from brain–body scaling relationships, such that an EQ greater than 1.0 means that the animal has a bigger brain than would be expected for an animal with a body of that size [91]. Although Jerison initially proposed EQ as an estimator of cognition, or “total information-processing capacity” [92], it has since been recognized that EQ comparisons across taxonomic groups have several severe limitations [93,94]. In particular, brain–body scaling varies among different taxonomic groups [95,96] with cetaceans being particularly deviant from general mammalian trends [97]. Connor et al. [98,99,100] acknowledged this problem and consequently restricted brain size comparisons to odontocetes of similar body size (showing interesting and marked differences among taxa). New data also demonstrate substantial variation in neuron densities across different taxonomic groups [101]. Therefore, because the same volume of brain does not necessarily indicate a similar number of neurons or synapses [94], this also renders simple metrics of brain size such as EQ incomparable across different taxonomic groups (see also [102]).

Secondly, it is naïve at best and disingenuous at worst to suggest that morphological descriptions of an animal’s brain can, in themselves, lead to reliable conclusions about that animal’s cognitive capacities. Even with a detailed description of an animal’s brain structures and cellular organization, the possible mapping between neural structures and behavior is not one-to-one [103]. For example, in different species, a particular neural sub-circuit might generate different behaviors, and a particular behavior might result from different neural configurations. Indeed, Marino herself made a similar point about possible variability in the neural underpinnings of cognitive capacities in a previous paper [104], noting that “*a compelling question for future study… is how such dissimilar neocortical cytoarchitectural motifs, such as that found in cetaceans and primates, result in convergent cognitive and behavioral characteristics*” (p. 1151). In other words, given that behavioral data suggested that cetaceans and primates may have some similar cognitive characteristics, the interesting question was how such similar cognition could arise from brain circuitry that was so different.

#### 2.4.2. Survivorship and Life Expectancy

In their discussion of survivorship and life expectancy, Marino et al. argue that (a) prior to 1992, “*captive orcas clearly did not live as long as free-ranging orcas (Small and DeMaster, 1995; Woodley et al., 1997)*” (p. 73); (b) a recent study [40] noted that although survival in facilities had improved, survival to age milestones “*remained poor compared to free-ranging orcas*” (p. 73); (c) calculations showing that the average life expectancy of captive-born killer whales numerically exceeds the average life expectancy in free-ranging populations [41] should be considered invalid because killer whale survival rates violate an assumption of that calculation; and (d) “*The high number of calf mortalities*” in facilities is “*alarming*” (p. 78). Importantly, there are at least three clear problems with these claims:

First, Marino et al. selectively apply validity criteria to calculations in different studies. That is, they correctly note that the equation used for converting Annual Survival Rate (ASR) to median life expectancy in Robeck et al. [41] assumes a constant survival rate for all animals in the calculation, which is violated by both historical and age-related changes in mortality [38,105]. However, this exact same assumption is also required for the calculation of ASR in the studies that analyzed survival data prior to 1992 [38,39], which they accept without question. It is unclear to what extent this violation affects the results of these calculations [105]; however, one must apply the criterion consistently. Since the same assumption is violated in both sets of studies, either both results must be accepted or both must be rejected.

Secondly, in stating that survival to age milestones was higher in free-ranging than in captive killer whales, Marino et al. failed to explain that the manner of calculating survival to age milestones differed dramatically between the two groups; that is, although the studies of wild killer whales included calves starting at 0.5 years of age [49,50], the study of captive killer whales [40] included calves from birth (including stillbirths). Note that calf mortality within the first 6 months of life in these wild populations is substantial. Olesiuk et al. [49] estimated it to be 37–50%. Olesiuk et al. [50] suggested that this may have been an overestimate, but noted that they still suspected that neonate mortality was “high.” Bain [106] independently estimated neonate mortality at 42% for part of that same population. The exclusion of young calves from one group but not the other would therefore clearly affect the results, making these groups noncomparable.

Thirdly, Marino et al.’s conclusion that “*the high number of calf mortalities*” in facilities is alarming (p. 78) is simply unfounded, given that (a) nowhere in their paper do they present any data or discussion regarding calf mortalities in facilities; and (b) the study they cited that analyzed calf mortality rate in facilities [40] found that 74% of all captive-born calves survived the first 6 months (i.e., a calf mortality rate of 26%). Referencing the same statistics cited above for wild populations, this study concluded that “*Thus it appears that captive-born calf mortality within the first 6 mo is generally consistent with observations of wild killer whale calf mortality.*” [40] (p. 12).

#### 2.4.3. Causes of Illness and Death

In their discussion of causes of death, Marino et al. argue that (a) “*Despite veterinary care and therapeutic intervention*,” killer whales in US marine theme parks have died from a number of diseases, “*including eight who reportedly died of pneumonia; three of encephalitis; three of bacteremia; and one of leptomeningitis*” (p. 72); and that (b) “*The frequency and nature of the diseases afflicting captive orcas… are largely distinct from their free-ranging counterparts*.” (p. 78). Marino et al. present this discussion as evidence that killer whales in facilities suffer from poorer health than free-ranging killer whales. However, there are at least three clear fallacies in this argument.

First, it is disingenuous to suggest that access to medical care should eradicate death by disease. In 2017, for example, over 49,000 people died from pneumonia in the United States [107]. Without some sort of rate comparison (that is, comparing the proportion of deaths from disease versus the proportion of deaths from other causes), the fact that deaths from diseases occur is not at all surprising, and says nothing about the quality of medical care involved. All animals die of something. To know whether these deaths are the result of poor welfare would require an analysis of relevant contextual factors, such as animal age, proportion of animals affected, and so forth.

Secondly, Marino et al. present no data to support their claim that the frequency of diseases is different in human care than in the wild. Instead, they present detailed information on causes of illness and death in captive killer whales, and then stipulate that this is different than in wild killer whales without providing evidence that it is. In fact, disease also occurs in wild killer whales [108,109,110,111]. However, causes of death in the wild and in captivity are difficult to compare, as we only rarely have post mortem examination of wild ones. Given that we have complete records for killer whales in facilities and incomplete records for killer whales in the wild, there is a clear difference in knowledge of the health issues for captive versus free-ranging killer whales. However, a lack of knowledge regarding disease prevalence in the wild should not be confused with a lack of diseases.

Thirdly, if the disease profile does differ between captive and free-ranging populations, that would only show that these environments are different—not that one environment is inherently worse than the other. Even if it turns out that there are health situations that are only encountered in facilities, there are others, such as starvation, ship strikes, and entanglement [112,113], that are only encountered in the wild.

#### 2.4.4. Space

Marino et al. [31] argue that housing killer whales in a contained space necessarily leads to chronic stress, stating that “*Abundant evidence shows that when a species is physiologically adapted to large home ranges, the opportunity to travel is critical to the health and well-being of its members (Clubb and Mason, 2003; Mason, 2010; McPhee and Carlstead, 2010)*” (p. 76). To be clear, we agree that adequate space to meet an animal’s needs is of course an important welfare consideration. So the relevant question, shared for all animals under human care, is what counts as adequate space. Rather than engaging in this question thoughtfully, however, Marino et al.’s discussion of the issue overreaches in their extrapolation of the evidence and oversimplifies the relevant causal factors, both of which impede a clear understanding of the evidence and possible courses of action to ensure optimal welfare.

Importantly, the references that Marino et al. cite note that “*Exactly how much space an animal needs is unclear, particularly because the critical factor for well-being is often the quality of the space*” [114] (p. 306) and that there is “*enormous variation between species in how they react to captivity*” [115] (p. 713). Ideally, then, the best evidence for determining space requirements for any particular species would come from studies of how the size, quality, and management of their living space impacts various welfare outcomes for that species. To our knowledge, no such study exists for killer whales. In lieu of these data, Marino et al. base their conclusions on studies showing that among terrestrial Carnivora (e.g., cats, bears, etc.), species with larger home ranges have higher infant mortality and more stereotypical pacing in zoos than species with smaller home ranges [116,117]. Unfortunately, they do not address the fundamental issue of whether these results can legitimately be extrapolated from terrestrial Carnivora to killer whales. This is a crucial omission, given that there are important reasons to believe that they cannot.

In the process of scientific extrapolation, one begins with knowledge of a causal relationship in one population, and attempts to draw an inference concerning that same relationship in a separate population [118]. To the extent that these populations are sufficiently similar, this extrapolation is generally viewed as justified. To the extent that these populations differ, however, the legitimacy of this extrapolation becomes more tenuous, especially if the populations differ with respect to causally relevant factors. In the case of terrestrial Carnivora versus killer whales, the populations differ in at least three meaningful respects: (1) biological order, (2) ecological niche, and (3) zoological practices relevant to providing physical exercise. The first two of these factors deal with the groups’ evolutionary histories, which can be expected to give rise to hardwired differences in physical needs and behavioral patterns. This is why Mason [115] notes that “*In captivity, being wide-ranging predicts poorer welfare in carnivores* (emphasis added)… *However, it remains unknown whether being migratory is a consistent risk factor for poor captive welfare*.” (p. 718). The third factor acknowledges that it is standard practice for killer whales in zoological settings to engage in positive reinforcement training sessions that ensure they get ample amounts of physical exercise. Although in recent years zoos have also begun to utilize positive reinforcement training with terrestrial animals for medical, husbandry, and enrichment purposes [119,120], there is as yet no similarly standard exercise program for terrestrial carnivores. The importance of this factor was discussed by McPhee and Carlstead [114], who noted that a multi-institutional study of polar bears [15], which have the largest home ranges of all carnivores and thus presumably should need the most space in zoos, found that polar bears who received training and more enrichment performed less stereotypical pacing than polar bears who received no training and less enrichment. Thus, the size, quality, and behavioral opportunities of a living space all play a role in determining what constitutes sufficient space for optimal welfare [114,121].

#### 2.4.5. Stress

**Figure 1:**
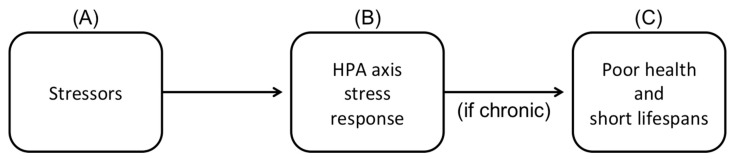
Schematic depiction of the causal chain leading from stressors to stress response to health effects.

In describing “*The association between stress, disorder, and death*” (p. 74), Marino et al. [31] note that (a) “*When the brain detects a threat, a coordinated physiological response … is activated, largely dependent upon the hypothalamus-pituitary-adrenal (HPA) axis*” (p. 74); (b) “*Although the HPA axis is critical to survival, its chronic activation and dysregulation can be detrimental to health and well-being*” (pp. 74–75); and (c) “*Maladaptive stressors…can lead to dysregulation of the HPA axis and related health effects*.” (p. 75). In other words, as depicted in Figure 1, what Marino et al. call “maladaptive stressors” lead to the activation of the HPA axis stress response, which, if chronic, leads to poor health and shortened lifespans. Marino et al. argue that this process describes the situation inherent in captive killer whales.

To properly evaluate this argument, one must first clarify and distinguish several concepts that are apparently conflated in Marino et al.’s discussion. First, as they note, a “stress response” refers to the physiological reactions of the body that are triggered when faced with a situation (a “stressor”) requiring some sort of action to mediate the impact of that stressor and return the animal to homeostasis (i.e., maintaining the relatively stable conditions necessary for survival and well-being) [122,123,124,125]. These stressors can be either things in the environment (e.g., a predator) or internal to the animal (e.g., hunger). In the short-term, the stress response is typically adaptive, helping the animal cope with internal and external perturbations to its normal, balanced state. However, if this physiological stress response goes on too long (i.e., becomes chronic), then this leads to detrimental effects on the animal, and becomes maladaptive [122,123,126]. In other words, it is the response to the stressor that is either adaptive or maladaptive. There is no such thing as a “maladaptive stressor”.

Secondly, it is worth noting that a measured physiological stress response is often the same whether the animal experiences the stressor as pleasant (eustress) or unpleasant (distress) [43,127,128]. The mammalian stress response readies the body for action in both aversive situations (e.g., fighting) and rewarding situations (e.g., play, mating). However, since the examples of “maladaptive stressors” provided by Marino et al. are all negative or unpleasant states (e.g., sensory overload, boredom), it may be that by “maladaptive”, they meant to indicate a stressor that the animal experiences as aversive.

With this tweak (or if we simply remove the term “maladaptive”), Marino et al.’s description of the HPA stress response and its effects is entirely non-controversial, as a number of studies have demonstrated different aspects of this causal chain in humans and many other animals (e.g., [129,130,131]). For purposes of completeness, it may be worth noting that an animal’s overall stress response also encompasses a number of additional effects that are not mediated through the HPA axis [122,124,129], and that the stress responses of terrestrial and marine mammals diverge in a number of interesting and important ways [129]. However, the HPA axis seems to function similarly in both groups [129]. Nonetheless, providing a detailed description of the biology of one part of the stress response is not the same thing as showing that the animals in question are experiencing these effects. Indeed, Marino et al. fail to provide evidence for any step of this process. There are no studies cited showing that killer whales in facilities show an elevated stress response as evidenced by cortisol levels, analyses of ACTH, or any other stress biomarkers relevant to the HPA axis in killer whales (Figure 1B). Marino et al. do cite a single study that showed elevated cortisol levels in bottlenose dolphins during a day with unusual loud noise and vibrations from drilling the walls surrounding the dolphins’ pools [132]. However, the point of that study was to test cortisol levels due to “the acute stress response” of this unusual situation compared to “regular days in the dolphinarium”. Marino et al. fail to note that multiple studies have shown that baseline cortisol levels in captive bottlenose dolphins are no higher than in their wild counterparts [133,134,135]. Nor do they provide credible evidence that killer whales in facilities today have poorer health or shorter lifespans than killer whales in the wild (Figure 1C), as discussed earlier. Instead, Marino et al. spend a lot of time discussing features in the killer whales’ environment that they describe as causing stress (by which they seem to mean both aversive and inducing a stress response). However, in addition to the fact that their descriptions of these potential stressors often take the form of emotive interpretations of unsubstantiated effects on the whales (e.g., “*sensory overload”*, “*sensory deprivation”*, “*boredom”*) rather than neutral descriptions of the actual environment, they also provide no evidence that the whales experience these aspects of their environment as stressful (i.e., causing a physiological stress response) or even aversive (in the sense that they find it unpleasant or seek to avoid it).

## 3. Conclusions

In conclusion, the creation of good animal welfare-related practices and policies for any species are dependent on valid scientific information. Ideally, this information includes data on the behavioral, cognitive, physiological, and social characteristics of the species, along with known welfare outcomes for different environmental, veterinary, and behavioral practices relevant to their care. Because this information, by its very nature, will initially be spread among numerous scientific publications spanning a number of different disciplines, the creation of a credible, comprehensive review that gathers, explains, and contextualizes this body of knowledge can be an invaluable tool for informing discourse and decision-making relevant to these animals. Of course, to be credible and useful, such reviews must present the data in an objective and reliable manner, taking care to clearly communicate what is known, what is not known, what conclusions can be supported by the data, and what areas we are lacking the data needed to draw reliable conclusions.

The topics raised in Marino et al.’s [31] review—e.g., life expectancy, stress, space, and whether the animals get enough physical, cognitive, and social simulation—are legitimate welfare issues for any animal under zoological care [45,136]. Unfortunately, Marino et al.’s discussion of these topics falls far short of the kind of accurate and unbiased review of research needed to reliably inform public discourse and decision-making about best welfare practices for killer whales. Instead, the pervasive problems with flawed and misleading referencing, interpretation, and argumentation throughout Marino et al.’s [31] paper make it impossible to determine the true state of knowledge of the issues raised, including whether there is reliable evidence regarding negative welfare indicators for killer whales, in which areas, and how best to address them.

Finally, the issues raised by our critique of Marino et al. [31] go beyond the discussion of zoological practices regarding killer whales. The state of the scientific knowledge of a given topic is relevant to legislative, regulatory, and policy decisions in many areas [16,21,24]. It is therefore incumbent upon scientists and science communicators to represent that scientific knowledge as completely, accurately, and objectively as possible [16,21]. Misrepresentations of the information will lead to a biased and incorrect body of knowledge that, instead of informing advancements, will impede productive discourse and may ultimately result in misinformed, arbitrary, or even harmful decisions.
